# Signal-to-Noise Efficiency Explains Inter-Observer Variability in Orientation Discrimination

**DOI:** 10.3390/vision10010004

**Published:** 2026-01-29

**Authors:** Thiago P. Fernandes, Natanael A. Santos, Linnea N. Dahlgren

**Affiliations:** 1 Perception, Neuroscience, and Behaviour Lab, Federal University of Paraiba, Joao Pessoa 58051-900, Brazil; 2 Department of Psychology, Federal University of Paraiba, Joao Pessoa 58051-900, Brazil; 3 Institute of Neurology, Ixelles, 1050 Brussels, Belgium

**Keywords:** orientation discrimination, visual noise, retinal eccentricity, perceptual variability, sensory encoding models

## Abstract

Background: Orientation discrimination tasks provide a core measure of visual sensitivity and are widely used to study how perceptual performance varies with stimulus uncertainty and visual field location. Here, we examined how external noise, retinal eccentricity, and individual perceptual efficiency shape orientation discrimination thresholds. Methods: Forty-two adults (mean age = 32.35 years, SD = 7.23) completed a two-alternative forced-choice task judging the orientation (clockwise vs. counterclockwise) of briefly presented Gabor patches under varying levels of external noise (low, medium, high) and eccentricity (0°, 5°, 10°). Orientation offsets ranged from −8° to +8°. Thresholds were estimated using psychometric functions and analyzed via rm ANOVA, linear mixed-effects models, and supervised machine learning. Results: Accuracy declined with increasing noise (ω^2^ = 0.48, *p* < 0.001) and improved with larger orientation offsets (ω^2^ = 0.62, *p* < 0.001). Thresholds increased with both noise (ω^2^ = 0.31, *p* = 0.002) and eccentricity (ω^2^ = 0.27, *p* = 0.003). Signal-to-noise efficiency was the strongest predictor (β = −0.72, *p* < 0.001); age alone was nonsignificant, but its interaction with eccentricity showed selective peripheral declines. Mixed-effects models confirmed spatial effects (β = 0.058, *p* < 0.001) and residual between-subject variability (σ^2^ = 0.14). Predictive models generalized well (R^2^ = 0.54). Conclusions: Orientation discrimination is shaped by both stimulus-level difficulty and individual differences in perceptual efficiency, which account for variability in sensitivity across visual conditions. Age-related differences emerge primarily under spatial load and depend on interactions between observer traits and task demands.

## 1. Introduction

Orientation discrimination is a fundamental measure of visual sensitivity, capturing how precisely observers can resolve small angular differences between visual stimuli. This task has long served as a model system in vision science, offering insights into sensory encoding, internal noise, and decision-making under uncertainty [1,2,3]. Although orientation discrimination is a well-established measure of visual sensitivity, recent work has highlighted systematic variability in performance arising from both stimulus context and observer-specific factors [4,5].

Two well-established determinants of orientation discrimination thresholds are external visual noise and retinal eccentricity. Thresholds increase under high noise, reflecting degraded signal fidelity and heightened uncertainty [6]. Similarly, orientation sensitivity declines with increasing eccentricity, consistent with anatomical and functional changes across the visual field, including reduced cortical magnification and increased receptive field size [7]. While these stimulus-driven effects are robust and widely replicated, they offer limited insight into how perceptual sensitivity varies across individuals under identical viewing conditions [8].

Research on orientation discrimination shows that stimulus properties like external noise, retinal eccentricity, and contrast, systematically modulate perceptual thresholds, reflecting well-defined limits on spatial resolution and signal fidelity [6,7,9]. These findings offer specific insights into how visual sensitivity also depends on external constraints [10]. Considering many psychophysical studies use repeated-measures designs under controlled conditions, individual variability is often treated as noise and averaged out. Few studies explicitly examine whether such differences reflect stable, trait-like aspects of perceptual processing, or assess their predictive value across varying visual demands [11,12]. Some recent work has begun to address this question, linking individual differences in perceptual thresholds to variation in sensory encoding and internal noise using neural and computational approaches [8,13]. By systematically varying both noise and eccentricity, this study evaluates whether individual differences in orientation sensitivity reflect generalizable traits rather than condition-specific performance.

We tested whether orientation discrimination performance reflects not only task difficulty but also stable, observer-specific patterns. We expected thresholds to increase with noise and eccentricity, consistent with established perceptual limits. More critically, we examined whether individual sensitivity profiles remained consistent across conditions and whether baseline performance generalized to more challenging visual contexts. Using a design that systematically varied stimulus uncertainty and retinal location, we combined psychophysical threshold estimation with traditional statistical methods and supervised machine learning to model individual performance across conditions. This integrative approach allowed us to evaluate whether performance variability is better explained by stable traits than by population-level averages, and to assess the external validity of these traits using cross-validated predictive models.

## 2. Materials/Subjects and Methods

Forty-two adults (N = 42; mean age = 32.35 years, SD = 7.23) participated in the study. All participants reported normal or corrected-to-normal vision and provided informed consent prior to participation. The experimental protocol was approved by the local ethics committee and conducted in accordance with the Declaration of Helsinki.

### 2.1. Apparatus and Stimuli

Stimuli were displayed on a gamma-corrected LCD monitor (ASUS ROG Swift PG259QN, ASUSTeK Computer Inc, Taipei, Taiwan; 24.5″ diagonal; native resolution 1920 × 1080 pixels; refresh rate 120 Hz). The display was calibrated using a Konica Minolta CS-100A photometer (Konica Minolta Inc., Tokyo, Japan), ensuring linear luminance output across the full dynamic range. At a viewing distance of 100 cm, the display subtended approximately 30.3° × 17.4° of visual angle, yielding a spatial sampling density of approximately 63 pixels per degree along the horizontal axis.

A spatially uniform mid-grey background (50 cd/m^2^) was presented throughout all trials. Stimuli consisted of static Gabor patches defined by a sinusoidal luminance grating with a spatial frequency of 3 cycles per degree and a fixed Michelson contrast of 70%, windowed by a circular, isotropic Gaussian envelope (σ = 0.7°). This resulted in an effective stimulus diameter of approximately 3° of visual angle.

The Gabor stimulus was mathematically defined as:

G(x,y,θ)=exp(−2σ2x′2+y′2)cos(2πfx′+ϕ)

where $x^{\prime }=xcos(\theta )+ysin(\theta )$ and $y^{\prime }=-xsin(\theta )+ycos(\theta )$ are rotated coordinates, $\sigma =0.7^{\circ }$ is the Gaussian envelope standard deviation, f=3 cycles/degree is the spatial frequency, ϕ=0 is the initial phase, θ denotes stimulus orientation, *cos* represents the grating, and *2πfx′* defines the spatial phase of the grating.

External sensory uncertainty was manipulated by adding zero-mean Gaussian luminance noise to the stimulus region. Three noise levels were defined by multiplicative scaling factors (0.5, 1.5, and 3.0) applied to the noise distribution, corresponding to low, medium, and high external noise conditions. Noise parameters were held constant across eccentricities to allow independent assessment of spatial and noise-related effects on orientation discrimination. Noise levels were implemented as relative scaling factors applied to the Gaussian noise distribution rather than absolute luminance units, a standard approach in external noise paradigms.

Stimuli were presented at retinal eccentricities of 0°, 5°, and 10° along the horizontal meridian while observers fixated a central black cross that remained visible throughout each trial. To minimize head movements and ensure stable viewing geometry, observers were seated with their head stabilized using a chin rest and headrest and were instructed to maintain fixation during stimulus presentation.

Prior to the main experiment, observers completed a short practice block comprising approximately 5% of the total number of trials to familiarize themselves with the orientation discrimination task and fixation requirement. To monitor task engagement, suprathreshold trials with large orientation offsets (±8°) were randomly interleaved throughout the experiment and comprised approximately 8% of total trials. Performance on catch trials was near ceiling across observers, indicating sustained task engagement and compliance.

### 2.2. Task and Procedure

Participants performed a two-alternative forced-choice (2AFC) orientation discrimination task designed to assess perceptual sensitivity to subtle deviations from vertical orientation under varying levels of external sensory noise.

Each trial began with a central fixation cross presented for 500 ms, followed by a brief stimulus presentation (100 ms) of a single Gabor patch at a predefined retinal eccentricity. The stimulus was oriented slightly clockwise (CW) or counter-clockwise (CCW) relative to the vertical axis. Stimulus position was balanced across left and right visual hemifields to control for potential lateralization effects. Participants indicated the perceived tilt direction by pressing one of two designated keys (left arrow for CCW, right arrow for CW). The response window was self-paced, and no trial-by-trial feedback was provided to minimize strategic adjustments or learning effects.

An inter-trial interval (ITI) followed each response, during which the fixation cross remained on screen to encourage gaze stability. The ITI was jittered between 400 and 800 ms to reduce temporal predictability and discourage anticipatory responses.

**Figure 1:**
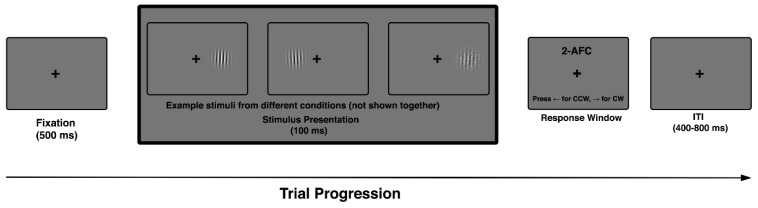
Trial sequence in the 2AFC orientation discrimination task. Each trial began with a 500 ms fixation cross, followed by a single Gabor stimulus (100 ms) with variable orientation, noise level, and eccentricity. Participants indicated whether the stimulus was tilted clockwise (CW) or counterclockwise (CCW). Example Gabors shown here illustrate stimulus variation across trials. Trials ended with a jittered inter-trial interval (400–800 ms), during which fixation was maintained.

The task included all combinations of external noise level and retinal eccentricity, with conditions fully randomized across trials. Orientation offsets were sampled from a fixed, symmetric set centered on vertical (e.g., ±2°, ±4°, ±6°, ±8°) and presented under varying levels of externally imposed noise, allowing precise estimation of discrimination thresholds across difficulty levels. Each participant completed a single experimental session lasting approximately 30–40 min, comprising multiple task blocks, with all combinations of noise level and eccentricity fully randomized across trials (Figure 1).

### 2.3. Psychometric Estimation and Signal-to-Noise Framework

Orientation discrimination performance was quantified using psychometric function analysis. For each participant and experimental condition, trial-level binary responses (clockwise vs. counterclockwise) were fitted with cumulative Gaussian psychometric functions to characterize the relationship between orientation offset magnitude and response probability. The threshold parameter (σ), corresponding to the standard deviation of the fitted function, was treated as the primary inferential index of perceptual sensitivity, while the bias parameter captured systematic response tendencies independent of sensitivity. The bias parameter was included to control for potential asymmetries in response preference and was evaluated descriptively; bias estimates were not treated as inferential outcomes and were not included as predictors in subsequent analyses.

Within an equivalent-noise and signal-detection framework, psychometric thresholds reflect the combined influence of stimulus-imposed external noise and observer-specific internal noise on the decision variable [14,15,16]. Accordingly, increases in threshold magnitude indicate reduced perceptual sensitivity, arising from higher effective noise, reduced signal utilization, or a combination of both.

Signal-to-noise ratio (SNR) was defined exclusively at the stimulus level based on physical stimulus parameters. Specifically, SNR was operationalized as a stimulus-defined ratio between orientation offset magnitude (signal strength) and externally imposed noise amplitude, and was therefore fixed by experimental design rather than estimated from behavioral responses [17]. Stimulus-defined SNR varied across experimental noise conditions but did not constitute an individual-level measure. Efficiency-related measures were instead inferred from threshold behavior across noise conditions, consistent with equivalent-noise formulations, and capture how effectively observers utilized available signal under increasing external noise.

SNR was used descriptively to characterize the sensory environment under which perceptual decisions were made, but was not treated as an independent behavioral outcome or inferential predictor of psychometric thresholds. Because both stimulus-defined SNR and psychometric thresholds are influenced by internal noise within a signal-detection framework, these quantities are not statistically independent. Accordingly, their relationship is interpreted as reflecting shared structure in the perceptual decision process rather than causal or mechanistically independent effects.

Throughout all analyses, psychometric thresholds were treated as the sole inferential index of perceptual sensitivity. Response accuracy was examined descriptively to characterize overall task engagement and relative stimulus difficulty, while stimulus-defined SNR was used solely to contextualize the physical signal environment under which perceptual decisions were made.

### 2.4. Statistical Analysis

All analyses were conducted in R (v4.5.1) and MATLAB (R2023b, MathWorks). Prior to statistical testing, data were screened for normality, extreme values, and outliers identified via Monte Carlo simulation. Descriptive statistics were computed to characterize overall task performance, stimulus difficulty, and participant engagement (Section 3.1).

Orientation discrimination thresholds were estimated for each participant and noise condition by fitting cumulative Gaussian psychometric functions to trial-level responses. The threshold parameter (σ) served as the primary index of perceptual sensitivity, while the bias parameter indexed systematic response preference.

Repeated-measures ANOVAs (rmANOVA) were used as the primary inferential framework to assess the effects of external noise level, retinal eccentricity, and orientation offset on psychometric discrimination thresholds (Section 3.2 and Section 3.3). Greenhouse–Geisser correction was applied when necessary. Effect sizes were reported as omega squared (ω^2^) with bias-corrected and accelerated (BCa) 95% confidence intervals. Significant effects were followed by Holm-corrected post hoc tests, with Hedges’ g and BCa 95% CIs used to quantify pairwise differences.

## 3. Results

### 3.1. General Task Performance

Forty-two participants completed the orientation discrimination task (mean age = 32.35 years, SD = 7.23, range = 21.1–44.1). Overall task accuracy was high across participants (M = 0.84, SD = 0.03; 95% BCa CI [0.83, 0.85]), indicating that the task was well understood and yielded reliable performance estimates across the sample.

**Figure 2:**
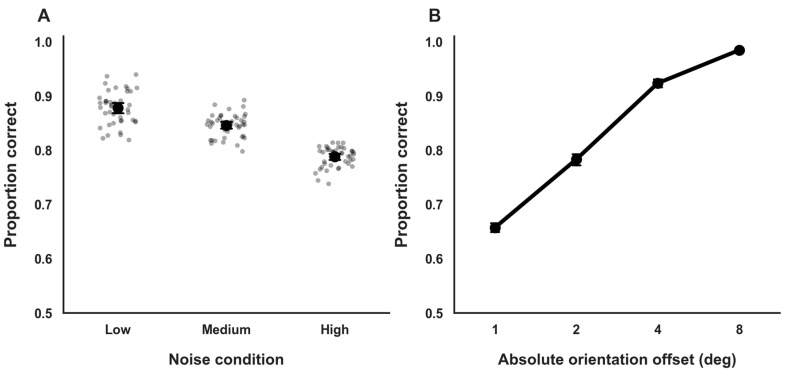
General Task Performance. (**A**). Orientation discrimination accuracy across external noise conditions. Dots represent individual participant means; circles and error bars indicate group means and 95% confidence intervals. (**B**). Accuracy as a function of absolute orientation offset, showing monotonic improvement with increasing stimulus strength. Error bars denote 95% confidence intervals.

Accuracy varied systematically across external noise conditions in a monotonic manner. Mean accuracy was highest under low external noise (M = 0.88, SD = 0.03; 95% BCa CI [0.87, 0.89]), intermediate under medium noise (M = 0.84, SD = 0.02; 95% BCa CI [0.83, 0.85]), and lowest under high noise (M = 0.79, SD = 0.04; 95% BCa CI [0.78, 0.80]; Figure 2A). This pattern is consistent with a gradual degradation of the stimulus signal-to-noise structure as external noise increased and serves as a descriptive manipulation check for the noise manipulation.

Performance also varied as a function of orientation offset magnitude. Accuracy was lowest for small, near-threshold orientation offsets (θ = ±1°; M = 0.65; 95% BCa CI [0.63, 0.67]) and increased steadily with larger offsets, reaching near-ceiling levels for clearly suprathreshold stimuli (θ = ±8°; M = 0.98; 95% BCa CI [0.96, 0.99]; Figure 2B). This monotonic pattern accords with the expected sigmoidal relationship between stimulus strength and response accuracy that underlies the psychometric function analyses used to estimate perceptual thresholds in subsequent sections.

Accuracy on suprathreshold trials was uniformly high (M = 0.98, SD = 0.01; 95% BCa CI [0.97, 0.99]; minimum participant accuracy ≥ 0.96), confirming sustained task engagement and minimal lapse rates throughout the experimental session. Accuracy measures in this section are reported descriptively to characterize task performance across stimulus difficulty levels and verify participant engagement. Inferential analyses of perceptual sensitivity are based exclusively on psychometric threshold estimates reported in subsequent sections.

### 3.2. Orientation Discrimination Thresholds as a Function of External Noise

**Figure 3:**
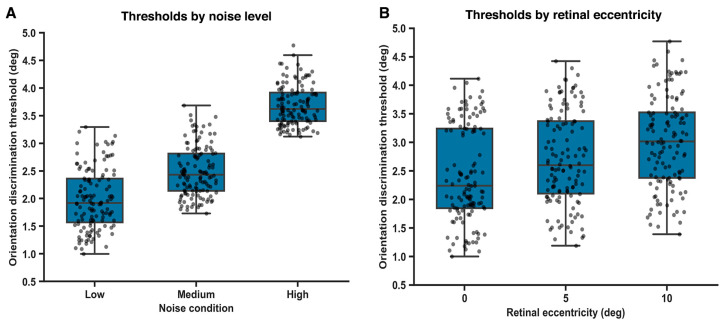
Effects of external noise and retinal eccentricity on orientation discrimination thresholds. (**A**). Orientation discrimination thresholds increase with external noise level. (**B**). Orientation discrimination thresholds increase with retinal eccentricity.

Orientation discrimination thresholds were systematically modulated by external noise level (Figure 3A). A within-subjects repeated-measures ANOVA revealed a significant main effect of noise condition on orientation thresholds, *F*(2, 82) = 12.6, *p* = 0.002; ω^2^ = 0.31, 95% BCas [0.18 to 0.44]. Post hoc comparisons (Holm-corrected) indicated that thresholds were significantly higher under high-noise conditions compared with both low-noise (*p* = 0.006; *g* = 0.92, 95% BCas [0.61 to 1.20]) and medium-noise conditions (*p* = 0.012; *g* = 0.65, 95% BCas [0.36 to 0.94]). The difference between low- and medium-noise conditions did reach statistical significance (*p* = 0.032; *g* = 0.38, 95% BCas [0.06 to 0.60]). These findings suggest a gradual reduction in orientation discrimination precision at intermediate levels of external noise, consistent with gradual noise-dependent degradation of perceptual fidelity rather than reflecting a categorical or abrupt change in threshold estimates.

### 3.3. Spatial Modulation of Orientation Discrimination

Orientation discrimination thresholds increased systematically as a function of retinal eccentricity (Figure 3B). A repeated-measures ANOVA revealed a significant main effect of eccentricity on orientation thresholds, *F*(2, 82) = 9.84, *p* = 0.003, with a moderate effect size (ω^2^ = 0.27; 95% BCas [0.14 to 0.38]).

Post hoc comparisons (Holm-corrected) indicated that thresholds were significantly higher at 10° eccentricity compared with both central (0°) presentation (*p* = 0.004; *g* = 0.88, 95% BCa [0.52 to 1.18]) and 5° eccentricity (*p* = 0.018; *g* = 0.61, 95% BCas [0.27 to 0.90]).

The difference between 0° and 5° eccentricity reached statistical significance but was comparatively smaller (*p* = 0.041; *g* = 0.36, 95% BCas [0.06 to 0.64]), indicating a gradual rather than abrupt decline in orientation discrimination sensitivity with increasing retinal eccentricity.

### 3.4. Latent Individual Modulators of Orientation Discrimination

To examine sources of inter-individual variability beyond task-defined manipulations, we analyzed differences in orientation discrimination thresholds remaining after accounting for experimental factors established a priori. External noise level and retinal eccentricity were treated as controlled task variables, as characterized in Section 3.2 and Section 3.3. The present analyses therefore focus on whether residual variability not explained by these manipulations exhibited systematic structure across participants, consistent with stable individual differences in perceptual sensitivity. Accordingly, we examined associations between psychometric threshold estimates and participant-level variables, with signal-to-noise efficiency included solely to contextualize individual differences within an equivalent-noise framework, rather than as an independent inferential outcome.

#### 3.4.1. Residual Inter-Individual Variability Assessed with Mixed-Effects Models

A linear mixed-effects model including external noise level and retinal eccentricity as fixed effects, with subject-specific random intercepts, was associated with a substantial proportion of variance in orientation discrimination thresholds. External noise exerted a strong effect on threshold magnitude (low vs. high noise: *β* = −1.761, SE = 0.053, *z* = −33.35, *p* < 0.001; medium vs. high noise: *β* = −1.228, SE = 0.053, *z* = −23.25, *p* < 0.001), and thresholds increased reliably with retinal eccentricity (*β* = 0.056°^−1^, SE = 0.005, *z* = 10.56, *p* < 0.001). Despite these robust task-level effects, the model revealed substantial unexplained between-participant variability, as reflected by a random-intercept variance of σ^2^_subject = 0.145 (SD = 0.38), indicating stable individual differences beyond task-defined manipulations.

To assess whether part of this residual variability exhibited systematic structure, age was subsequently added as a continuous covariate. Including age led to a reduction in between-subject variance (σ^2^_subject = 0.104; Δσ^2^ = 0.041), indicating that age was associated with a modest portion of the residual inter-individual variability beyond experimental factors. Age showed a small but statistically detectable association with threshold magnitude (*β* = 0.031, SE = 0.008, *z* = 3.72, *p* < 0.001), while the fixed effects of noise condition and eccentricity remained stable in both magnitude and significance. This pattern suggests that age-related variance reflects a secondary modulation of perceptual sensitivity rather than a primary determinant of task performance. By jointly modeling external noise and eccentricity as fixed effects and subjects as random effects, this approach provides a principled decomposition of task-driven and observer-specific sources of variance, without assuming independence of repeated observations.

#### 3.4.2. Exploratory Correlational Structure of Residual Threshold Variability

To further characterize residual structure, exploratory correlational analyses were conducted. When thresholds were collapsed across noise conditions, age was not significantly correlated with overall threshold magnitude (*r* = 0.14, *p* = 0.13). However, when examined separately by retinal eccentricity, age–threshold associations varied modestly with spatial demand (0°: *r* = 0.17, *p* = 0.054; 5°: *r* = 0.20, *p* = 0.027; 10°: *r* = 0.28, *p* = 0.001). These analyses were exploratory and hypothesis generating, included to examine whether residual variability exhibited systematic structure across spatial demands, and are interpreted cautiously.

#### 3.4.3. Conceptual Role of Stimulus-Defined Signal-to-Noise Ratio

Accordingly, we examined associations between psychometric threshold estimates and participant-level variables, with stimulus-defined signal-to-noise ratio included only as a descriptive quantity to contextualize performance within an equivalent-noise framework, rather than as an independent inferential predictor. As intended, SNR did not vary meaningfully across participants when averaged across conditions and therefore did not account for between-subject differences in residual threshold estimates. Associations between threshold magnitude and SNR are interpreted as reflecting shared dependence on internal noise within the perceptual system rather than independent or causal effects.

**Figure 4:**
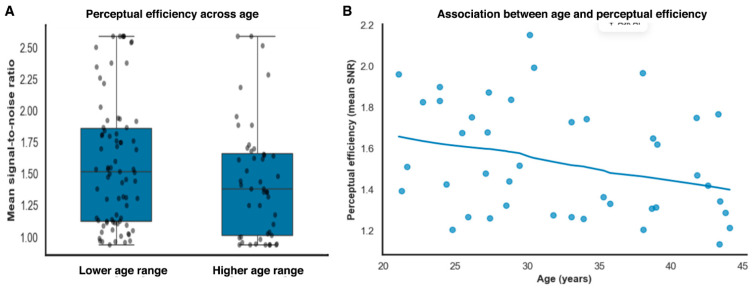
Age-related differences in perceptual efficiency. (**A**). Perceptual efficiency (mean signal-to-noise ratio) across age subdivisions identified using data-driven clustering, illustrating reduced efficiency in the higher age range. (**B**). Continuous association between age and perceptual efficiency, showing a gradual decline in mean signal-to-noise ratio with increasing age.

Signal-to-noise ratio showed a statistical association with threshold magnitude (*β* = −0.72, SE = 0.12, *t*(121) = −6.02, *p* < 0.001, partial *r*^2^ = 0.23, 95% BCas [−0.95 to −0.49]). Importantly, this association reflects shared variance arising from internal noise contributions common to both measures within an equivalent-noise framework, rather than an independent or causal effect of efficiency on threshold performance. In contrast, age did not contribute independently once perceptual efficiency and noise level were accounted for (*β* = 0.006, SE = 0.005, *t*(121) = 1.20, *p* = 0.23), indicating that age-related variance in thresholds was largely captured by task-dependent perceptual factors rather than reflecting a uniform decline in baseline sensitivity (Figure 4A).

### 3.5. Predictive Modelling of Individual Sensitivity

To evaluate the extent to which individual differences in orientation discrimination sensitivity could be predicted from task-relevant and demographic features, we implemented a supervised machine-learning framework with cross-validated performance assessment. Models were trained to predict subject-level orientation thresholds using age, retinal eccentricity, signal-to-noise efficiency, and their interactions as candidate predictors. Unlike ordinary regression, predictive modeling incorporated all task-defined factors and their interactions while respecting the multivariate structure of the data, providing an independent assessment of the predictability of individual differences.

Model performance was assessed using 10-fold cross-validation. Across folds, the predictive model showed robust generalization performance (mean cross-validated *R*^2^ = 0.54, SD = 0.12, 95% CI [0.31, 0.71]), indicating that more than half of the inter-individual variance in orientation discrimination thresholds could be explained in unseen data. In absolute terms, prediction error was modest relative to the observed threshold range (RMSE = 0.46°, SD = 0.09, 95% CI [0.33°, 0.61°]; MAE = 0.37°, SD = 0.08, 95% CI [0.25°, 0.52°]), demonstrating good quantitative agreement between predicted and observed thresholds. Model performance substantially exceeded that of a null baseline model predicting the mean threshold (*R*^2^ = 0.02, 95% CI [−0.04, 0.08]; RMSE = 0.98°, 95% CI [0.84°, 1.14°]), indicating that a substantial portion of individual differences in perceptual sensitivity can be predicted from a compact set of task-related features rather than reflecting idiosyncratic noise.

Permutation-based feature importance analyses converged closely with the inferential findings reported in Section 3.4. Signal-to-noise efficiency emerged as the strongest predictor of individual thresholds (normalized importance = 0.69, 95% CI [0.61, 0.78]), consistent with earlier regression and mixed-effects analyses identifying perceptual efficiency as a primary mediator of performance variability. Notably, the interaction between age and retinal eccentricity constituted the second most informative feature (importance = 0.58, 95% CI [0.47, 0.66]), exceeding the predictive contribution of either age or eccentricity considered in isolation. In contrast, the main effects of age (importance = 0.11, 95% CI [0.04, 0.19]) and eccentricity (importance = 0.06, 95% CI [0.01, 0.12]) contributed minimally once interaction terms were taken into account.

## 4. Discussion

Our findings showed that orientation discrimination performance was systematically modulated by external noise, retinal eccentricity, and orientation offset, replicating well-established effects on perceptual sensitivity. Thresholds increased with higher noise levels and greater eccentricity, and decreased with larger angular offsets, indicating that the task reliably captured graded variations in stimulus difficulty. Despite these strong task-level effects, substantial variability remained across participants, motivating an examination of individual differences beyond experimentally controlled factors.

The present findings can be interpreted within an equivalent-noise framework, in which perceptual thresholds reflect the interaction between stimulus-imposed external noise and observer-specific internal constraints [16,17,18]. By manipulating external noise and examining threshold behavior across noise regimes, the task characterizes how effectively observers utilize available sensory signal under increasing uncertainty [6]. Importantly, signal-to-noise ratio in the present study was defined at the stimulus level to characterize the physical sensory environment, whereas efficiency-related measures were derived from threshold behavior across noise conditions, providing a computational-level summary of internal constraints without constituting direct estimates of neural noise [3,18].

These findings suggest that perceptual sensitivity is influenced by both external and internal constraints, rather than being determined by fixed thresholds. The gradual declines in accuracy and increases in thresholds with added external noise align with models in which perceptual noise interacts with stimulus uncertainty, as described in signal detection theory [19,20] and Bayesian observer frameworks [21,22]. Similarly, the increase in orientation discrimination thresholds with retinal eccentricity is consistent with known limits of peripheral processing, including reduced cortical magnification and larger receptive fields [23,24], which diminish spatial resolution and contrast sensitivity.

A plausible physiological substrate for such internal constraints lies in the regulation of excitation–inhibition balance within early visual cortex. Perceptual precision depends on the coordinated interaction between excitatory drive and inhibitory control, which together shape neural gain and suppress response variability [25]. In human studies, individual orientation discrimination thresholds have been shown to correlate negatively with resting GABA concentration in visual cortex, and gamma oscillation frequency correlates positively with GABA levels, indicating that inhibitory tone plays a central role in maintaining signal fidelity. Although neurochemical measures were not obtained in the present study, these findings support the view that inter-individual differences in local circuit regulation, rather than fixed sensory limits, contribute to variability in perceptual sensitivity [26,27]

Importantly, the strong negative association between signal-to-noise efficiency and discrimination thresholds indicates that individual differences in perceptual encoding play a central role in shaping behavioral performance [4]. This finding reinforces computational accounts that emphasize internal noise and encoding efficiency over fixed capacity limits [28]. Age-related analyses were included to assess whether these efficiency-related effects remained robust after accounting for demographic variability, rather than to test age as a primary explanatory factor. Although modest age-by-eccentricity patterns were observed, these effects were secondary to the primary influences of noise and efficiency and are interpreted cautiously [29]. Notably, the age-by-eccentricity interaction identified by exploratory analyses and predictive models suggests that age-related variability emerges primarily under increased spatial demands, consistent with a modulatory role of age on peripheral processing rather than a global effect on perceptual sensitivity [30].

Previous work has documented the effects of noise and eccentricity on visual discrimination, but the present study extends this literature by isolating individual-level factors that account for threshold variability [31,32]. Across both mixed-effects and predictive modelling approaches, signal-to-noise efficiency consistently emerged as the strongest predictor of perceptual sensitivity, whereas age-related effects were spatially specific rather than global. Together, these findings indicate that perceptual variability reflects systematic differences in signal utilization rather than fixed sensory limits. Although the present results demonstrate that these individual differences generalize across task conditions and modelling frameworks, establishing reproducibility across independent measurements will be an important next step for confirming longer-term stability of efficiency-related measures [14].

The present study offers several methodological advantages, including a well-powered within-subjects design, fine-grained parametric manipulation of stimulus features, and the integration of both classical inferential statistics and predictive modelling. Psychometric threshold estimation, inclusion of catch trials to ensure attentional engagement, and robust cross-validation procedures contribute to the reliability and generalizability of the results. Nonetheless, certain limitations should be noted. The task employed brief, static stimuli in a controlled laboratory environment, which may not fully reflect the complexity of naturalistic visual processing. Although the sample spanned a broad adult age range, it did not include older adults beyond midlife or clinical populations, limiting extension to more diverse cohorts. Additionally, while the predictive models identified key behavioral predictors, they did not directly address underlying neural mechanisms.

Future research could examine how perceptual efficiency operates in more ecologically valid settings. Adapting orientation discrimination tasks to dynamic or multisensory contexts may clarify how perceptual strategies adapt to real-world demands. Extending the modelling approach across sensory modalities or cognitive domains could further test the generalizability of efficiency profiles beyond basic visual tasks. Finally, integrating behavioral data with neuroimaging or electrophysiological measures would enable more direct links between individual threshold variability and underlying neural architecture, advancing mechanistic accounts of perceptual traits.

This perspective advances a more individualized account of visual perception, emphasizing adaptive efficiency over fixed limits, and positioning threshold variability as a systematic, trait-like marker of perceptual strategy, one that can inform models of sensory encoding, age-related change, and broader theories of visual performance under uncertainty.

## Data Availability

The datasets generated and analyzed during the current study are available from the corresponding author upon reasonable request.
